# Therapeutic Efficacy of Endoscope‐Guided Coblation for Tongue Base Reduction as Part of Multilevel Surgery in Moderate to Severe OSA Patients

**DOI:** 10.1002/wjo2.70116

**Published:** 2026-05-21

**Authors:** Minju Kim, Siyeon Jin, Eun Hyun Cho, Dongyoung Kim, Doo Hee Han, Hyun Jik Kim

**Affiliations:** ^1^ Department of Otorhinolaryngology‐Head and Neck Surgery National Medical Center Seoul the Republic of Korea; ^2^ Department of Otorhinolaryngology‐Head and Neck Surgery Seoul National University College of Medicine, Seoul National University Hospital Seoul the Republic of Korea; ^3^ Sensory Organ Research Institute Seoul National University Medical Research Center Seoul the Republic of Korea

**Keywords:** coblation, endoscope‐guided tongue base reduction, obstructive sleep apnea, therapeutic efficacy

## Abstract

**Objective:**

Obstructive sleep apnea (OSA) frequently results from multilevel upper airway obstruction involving the palate, lateral walls, and tongue base. Among various techniques, endoscope‐guided coblation for tongue base reduction (TBR) provides precise resection with excellent hemostasis and minimal morbidity. This study aimed to evaluate the therapeutic efficacy and safety of endoscope‐guided coblation for TBR as a component of multilevel surgery in patients with moderate to severe OSA.

**Study Design:**

Case series.

**Setting:**

Single‐institution academic tertiary medical center.

**Subjects and Methods:**

The study included 92 OSA patients (85 males and 7 females) with identified tongue base (TB) narrowing in a single tertiary center. Patient demographics, subjective symptoms, polysomnography parameters, drug‐induced sleep endoscopy findings, positive airway pressure data, and postoperative complications following endoscopic coblation TBR were retrospectively reviewed.

**Results:**

The mean age of the patients was 40.8 ± 12.8 years, and the mean body‐mass index was 26.8 ± 4.0 kg/m^2^. The success rate of endoscopic coblation TBR was 56.1% in moderate or severe OSA patients, with 66.7% showing significant improvement in sleep parameters. The preoperative apnea‐hypopnea index (AHI) was 47.3 ± 22.2 events/hr, which decreased to 27.8 ± 23.8 events/hr postoperatively at 6 months. Supine AHI, apnea index (AI), and lowest oxygen saturation (SpO_2_) were also significantly improved. Sleep efficiency and REM percentage increased substantially. Postoperative complications were minimal, with pain being the most common complaint, which rarely lasted longer than a week.

**Conclusion:**

Endoscope‐guided coblation TBR, when incorporated into multilevel sleep surgery, significantly improves polysomnographic outcomes, including AHI, oxygen saturation, and sleep efficiency, while maintaining a favorable safety profile. This approach offers a practical, cost‐effective, and minimally invasive alternative, broadening access to effective upper airway surgery for OSA patients.

## 1 Introduction

Obstructive sleep apnea (OSA) occurs due to fixed or dynamic upper airway obstruction caused by anatomical factors or abnormal upper airway motor tone. Upper airway obstruction can result from the collapse of single or multiple structures, such as the soft palate, tonsils, lateral pharyngeal walls, and base of the tongue [1, 2, 3]. The hypoxic events and oxidative stress resulting from reduced or completely stopped airflow in OSA are associated with significantly increased morbidity and mortality, including a heightened risk of cardiovascular events if not properly treated [4, 5]. Positive airway pressure (PAP) is the primary treatment for symptomatic or moderate to severe OSA. However, surgical interventions are frequently required for OSA treatment, particularly when the causes of upper airway narrowing are clear and when anatomical features significantly influence airflow during sleep [6, 7, 8]. Sleep surgeries for OSA aim to reduce the volume of pharyngeal structures that block the upper airway, to enhance stability of the pharyngeal airway, and to widen the pharyngeal lumen sufficiently. Many studies have demonstrated the clinical benefits of sleep surgeries, including improvement of sleep parameters and relief from subjective symptoms and life‐threatening conditions in OSA patients. Various surgical methods to treat OSA include maneuvers to widen the upper airway and correct excessive collapsibility of redundant tissues including the velum, oropharynx, tongue base, and epiglottis [9, 10].

Tongue base (TB) narrowing has been considered as a major anatomic factor inducing airway narrowing in OSA patients and can contribute independently to the pathogenesis of OSA combined with partial or complete obstruction at the palate level [11]. Also, airway narrowing at the retroglossal area is more prevalent in patients with severe OSA, and incomplete correction of TB narrowing in moderate or severe OSA is closely related with treatment failure of sleep surgeries with higher apneic events [11, 12]. Therefore, there is a need for more effective correction of TB narrowing and diverse surgical techniques for reducing TB volume have been introduced as components of multilevel surgery to improve the surgical outcomes of OSA.

TB resection (TBR) using endoscope‐guided coblation and robotic surgery supported by high‐quality visualization devices have been utilized to reduce TB volume in OSA patients [13, 14]. Endoscopic coblation has many benefits such as enhanced visibility during TBR, efficient tissue reduction, and better control of intraoperative bleeding [15]. Robot‐assisted TBR has been successfully applied to treat TB narrowing in patients with OSA, achieving a relatively fair success rate of 50%–70% [16, 17]. However, robot‐assisted TBR sometimes encounters spatial constraints in sleep surgery, leading to instrument collisions. In addition, during robot‐assisted TBR, a monopolar device is used to excise tongue base tissue; therefore, there are concerns regarding postoperative pain, bleeding, and delayed wound healing. Furthermore, cost–benefit issues also present challenges [18, 19].

This study aimed to evaluate the efficacy of endoscope‐guided coblation for TBR as part of multilevel surgery in OSA patients with identified tongue base narrowing and to determine whether this technique can significantly improve sleep parameters with minimal complications.

## 2 Methods

### 2.1 Participants

Ninety‐two patients with OSA who underwent endoscope‐guided coblation for TBR at our hospital between 2015 and 2021 were included in this study. The study was approved by the Institutional Review Board of Seoul National University Hospital, Seoul, Korea (IRB No. 2408‐033‐1558) and was conducted in accordance with the principles of the Declaration of Helsinki. All personal information was kept confidential as required and the requirement for informed consent was waived because of the retrospective nature of the study. All patients either failed to adhere to or refused PAP therapy. The criteria for considering TBR included moderate to severe OSA (apnea‐hypopnea index (AHI) greater than 15 events/hr) or a TB obstruction grade of 2 (greater than 75% narrowing) or higher as determined by drug‐induced sleep endoscopy (DISE). The VOTE classification was used to evaluate the sites and degrees of upper airway obstruction [20]. OSA patients included in the study had Grade 2 or higher narrowing at the level of the velum and oropharynx in addition to TB narrowing. Endoscope‐guided coblation TBR was performed in conjunction with other multilevel procedures as indicated, including septoturbinoplasty in 88 patients (95.7%), tonsillectomy in 75 patients (81.5%), uvulopalatal flap in 74 patients (80.4%), and relocation pharyngoplasty in 83 patients (90.2%).

**Figure 1:**
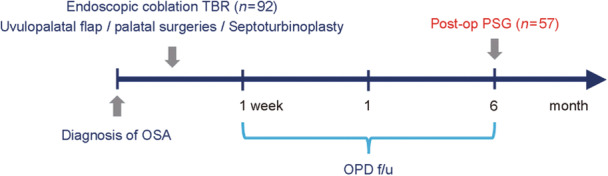
Schematic of the study design. OPD f/u, outpatient department follow‐up; OSA, obstructive sleep apnea; PSG, polysomnography; TBR, tongue base resection.

The demographic and anatomical characteristics of the patients were retrospectively reviewed, along with their pre‐ and postoperative polysomnographic (PSG) data. Age, sex, body weight, height, body mass index (BMI), modified Mallampati classification, and tonsil size were reviewed. Of the 92 patients included, 57 underwent postoperative PSG at 6 months after TBR. PSG parameters, including AHI, supine AHI, apnea index (AI) (events/h), lowest oxygen saturation (SpO_2_) (%), total sleep time (min), sleep efficiency (%), rapid eye movement (REM) sleep percentage (%), snoring, and the Epworth Sleepiness Scale (ESS), were compared before and following TBR in conjunction with nasal and palatal surgeries (Figure 1). Additionally, early or late complications, including pain, bleeding, and abnormal sensations, were recorded at the time of discharge and during subsequent outpatient visits, if any were present. Surgical success was defined according to Sher's criteria, as achieving a 50% reduction in the AHI and a postoperative AHI of less than 20, consistent with previous reports [21].

### 2.2 Surgical Technique for Coblation TBR

Preoperative upper airway evaluation, including DISE, was conducted to determine the degree and level of obstruction, which aided in selecting patients for TBR to enhance airflow in the retroglossal area. Tracheostomy was not routinely performed.

Patients were placed in the Rose position under general anesthesia with nasotracheal intubation. The nasotracheal tube was placed posterior to the surgical field and could be easily adjusted by the assistant if necessary. Examination of the TB was initially performed using a 30° endoscope, followed by placement of the McIvor mouth gag retractor (Sklar Instruments, PA, USA) to ensure a clear surgical field. The size of the blade was based on patient anatomy and sex, with an effort to use the largest blade possible. Anatomical landmarks, including intended surgical margins, were identified before proceeding. The coblator (Evac70, Smith & Nephew, London, UK) was then used to reduce redundant TB tissue under 30° endoscope guidance. The margin of resection extended anteriorly to the circumvallate papillae, medially to the midline in the vallecula, and laterally to the tonsillar fossae. Lymphoid tissue, including the lingual tonsil, predominantly was resected, with efforts to preserve muscle as much as possible. Care was taken to avoid injuring the branches of the glossopharyngeal nerve and the dorsal lingual artery. Hemostasis at the end of the procedure was achieved using the coblator coagulation mode. Patients were routinely discharged 2 days after surgery.

### 2.3 Statistical Analysis

Postoperative changes in PSG parameters, including AHI, supine AHI, AI, lowest SpO_2_ (%), total sleep time (min), sleep efficiency (%), REM sleep percentage (%), snoring, and ESS were assessed. Based on data normality, continuous data were analyzed using Student's t‐test or Mann–Whitney *U* test to compare variables in subgroups. Demographic data are presented using descriptive statistics. A p‐value less than 0.05 was considered statistically significant. Statistical analyses were performed using the IBM SPSS (version 27.0; SPSS Inc., IBM Corp, Armonk NY, USA) and GraphPad Prism 10 (GraphPad Software Inc., San Diego, CA, USA).

## 3 Results

### 3.1 Demographic and Anatomical Characteristics

The mean age of the patients was 40.8 ± 12.8 years, with a predominance of males (85 males and 7 females). The mean BMI was 26.8 ± 4.0 kg/m^2^, with 64.1% classified as obese (BMI ≥ 25 kg/m^2^) group. The mean AHI was 47.0 ± 21.6 events/hr, and the lowest SpO_2_ was 77.8 ± 8.6%. Total sleep time was 359.9 ± 83.9 min. The mean AI was 24.1 ± 22.6 events/h, and mean REM sleep percentage was 17.9 ± 11.3% (Table 1). The AHI was used to assess OSA severity. Of the 92 patients, 21 were classified as having moderate OSA, while 71 had severe OSA. The criterion for TBR was moderate to severe OSA based on AHI or a TB obstruction grade of 2 (indicating greater than 75% narrowing) or higher, as determined by DISE.

**Table 1:** Demographic and polysomnographic parameters of the patients (total *N* = 92).

Demographic and anatomical parameters	
Age (years)	40.8 ± 12.8
Male (%, *N*)	92.4% (85)
Female (%, *N*)	7.6% (7)
BMI (kg/m^2^)	26.8 ± 4.0
BMI ≥ 25 (%, *N*)	64.1% (59)
Tonsil grade (%, *N*)	
0	6.5% (6)
I	50.0% (46)
II	20.7% (19)
III	22.8% (21)
IV	0.0% (0)
Modified Mallampati grade (%, *N*)	
I	2.2% (2)
II	10.9% (10)
III	56.5% (52)
IV	30.4% (28)
Polysomnographic parameters	
AHI (/h)	47.0 ± 21.6
Lowest O_2_ saturation (%)	77.8 ± 8.6
Total sleep time (min)	359.9 ± 83.9
Apnea index (/h)	24.1 ± 22.6
REM sleep percentage (%)	17.9 ± 11.3

*Note:* Continuous variables were expressed as mean ± SD.

Abbreviations: AHI, apnea‐hypopnea index; AI, apnea index; BMI, body mass index; REM, rapid eye movement; SD, standard deviation.

### 3.2 Preoperative Versus Postoperative PSG Findings

For the 57 patients who underwent postoperative polysomnography 6 months after endoscopic coblation TBR, PSG parameters were analyzed (Table 2). The mean AHI for the entire group was 47.3 ± 22.2 (events/h) preoperatively and significantly reduced to 27.8 ± 23.8 postoperatively (*p* < 0.001). Supine AHI and AI significantly decreased from 66.3 ± 50.7 (events/h) to 35.2 ± 27.1 (*p* < 0.001) and from 27.4 ± 25.1 (events/h) to 15.3 ± 20.9 (*p* = 0.005), respectively. The lowest SpO_2_ significantly improved from 78.0 ± 8.3 to 83.1 ± 8.4% (*p* < 0.001). Total sleep time increased from 374.6 ± 78.2 to 397.1 ± 76.6 min, although this was not statistically significant (*p* = 0.130). Sleep efficiency improved from 84.8 ± 9.2 to 87.7 ± 9.0% (*p* = 0.042). The percentage of REM sleep increased from 16.9 ± 6.3% to 20.3 ± 6.9% (*p* = 0.003).

**Table 2:** Preoperative and postoperative polysomnographic parameters.

	Preoperative	Postoperative	*p*‐Value
AHI (/h)	47.3 ± 22.2	27.8 ± 23.8	< 0.001
Supine AHI (/h)	66.3 ± 50.7	35.2 ± 27.1	< 0.001
AI (/h)	27.4 ± 25.1	15.3 ± 20.9	0.005
Lowest SpO_2_ (%)	78.0 ± 8.3	83.1 ± 8.4	< 0.001
Total sleep time (min)	374.6 ± 78.2	397.1 ± 76.6	0.130
Sleep efficiency (%)	84.8 ± 9.2	87.7 ± 9.0	0.042
REM percentage (%)	16.9 ± 6.3	20.3 ± 6.9	0.003
Snoring	2.2 ± 0.8 moderate to severe	1.4 ± 0.7 mild to moderate	< 0.001
Epworth sleepiness scale	9.0 ± 4.7	8.1 ± 5.2	0.212

*Note:* Continuous variables were expressed as mean ± SD.

Abbreviations: AHI, apnea‐hypopnea index; AI, apnea index; BMI, body mass index; REM, rapid eye movement; SD, standard deviation.

### 3.3 Surgical Outcomes of Endoscopic Coblation TBR

**Figure 2:**
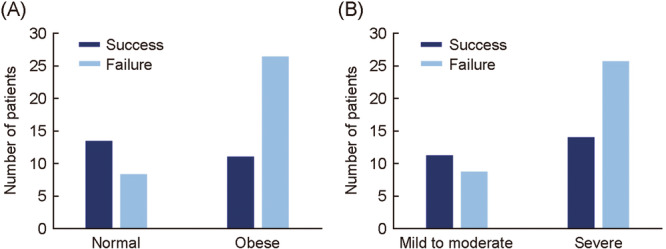
Success rate based on obesity (A) and the severity (B) of obstructive sleep apnea. Surgical success was defined as a 50% reduction in the Apnea‐Hypopnea Index (AHI) and a postoperative AHI < 20.

Therapeutic outcomes were evaluated via AHI after endoscopic coblation TBR. Given an arbitrary selection of 50% reduction in AHI and AHI < 20 as a threshold, 32 patients responded to surgery, yielding a sleep‐surgery success rate (including endoscopic coblation TBR) of 56.1% in OSA patients with TB narrowing. When improved cases, defined as a 25%–50% reduction in AHI, were added to the success cases, the overall response rate was 66.7% (38 of 57 patients). However, obese patients (BMI ≥ 25 kg/m^2^) were more likely to fail (odds ratio [OR] = 3.71, 95% confidence interval [CI] [1.17–11.73], *p* = 0.045). Patients with severe OSA tended toward poorer outcomes but did not exhibit any significant differences in outcomes compared to those with moderate OSA (OR = 2.23, 95% CI [0.69–7.11], *p* = 0.236, Figure 2). Tonsil or palatal grade did not show significant change between subgroups (Table 1).

**Figure 3:**
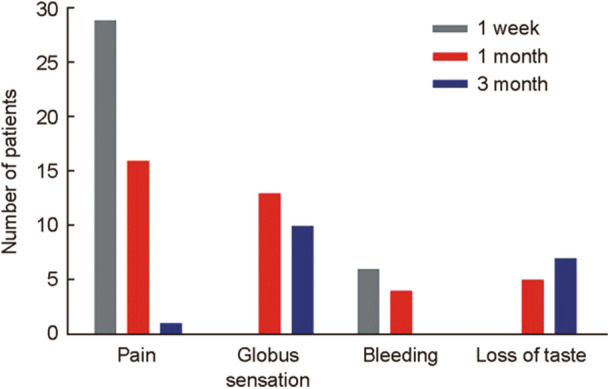
Subjective complaints and side effects observed 1 week, 1 month, and 3 months after surgery. Bars represent the number of subjects.

Potential complications, such as pain, bleeding, globus sensation, swallowing difficulty, and taste disturbance, were assessed at 1 week, 1 month, and 3 months postoperatively (Figure 3). Postoperative pain was the most common symptom, reported by 29 patients at 1 week postoperatively, but only one patient reported pain 3 months after surgery. We determined that in most patients who underwent endoscope‐guided TBR using coblation and palatal surgeries including tonsillectomy, the risk of persistent pain disappeared after 1 month or more after surgery. Additionally, 13 patients experienced globus sensation 1 month after surgery, with this number decreasing to 10 patients at 3 months postoperatively. Five patients complained of loss of taste 1 month after surgery. All five patients did not recover by 3 months, and additional two patients reported loss of taste 3 months after surgery. We estimated that loss of taste requires observation for at least 3 months, especially after coblation TB surgery, and may require medication in some cases. No major complications, such as massive bleeding at the level of TB and oropharynx or severe dyspnea to require tracheostomy, were observed.

## 4 Discussion

Our clinical findings showed that endoscope‐guided coblation for TBR should be considered an effective surgical option for widening the retroglossal area in patients with OSA and yielded favorable therapeutic outcomes in patients with moderate to severe OSA as part of multilevel surgery combined with nasal or palatal procedures.

Evaluation of the degree of narrowing or excessive collapse is necessary to determine appropriate surgical options in patients with OSA when repositioning or removing redundant upper airway tissues. In addition, the success rate of sleep surgery varies significantly depending on the anatomical site of airway obstruction [22, 23, 24]. The TB region has been considered as a clinically important anatomic structure in the pathogenesis of OSA and OSA patients with excessive TB narrowing exhibited higher AHI scores in concert with intermittent hypoxia and oxygen desaturation. However, it might be technically difficult to remove redundant tissues in the TB region and to improve TB narrowing through surgical resection in the OSA patients [25]. In addition, surgical correction of TB narrowing sometimes causes serious complications such as bleeding, dysphagia, or respiratory difficulties [26]. Consequently, there is a need for effective and safe surgical methods to correct TB narrowing and achieve satisfactory outcomes for OSA patients with retroglossal area collapse.

Robot‐assisted TBR, coblation TBR and hypoglossal nerve stimulation (HGNS) have been proposed to address retroglossal narrowing in OSA subjects [19, 27, 28, 29]. Although robot‐assisted TBR and HGNS have produced relatively good therapeutic outcomes in patients with OSA and retroglossal airway narrowing, these procedures are invasive and are often associated with significant postoperative pain and dysphagia. In addition, their high surgical costs may pose a burden to patients.

Our clinical data revealed a significant reduction in OSA patients following septoturbinoplasty, uvulopalatal flap, palatal surgeries and TBR using coblation and this substantial decrease underscores the effectiveness of this procedure in reducing apneic events. Additionally, there were significant reductions in supine AHI and AI and an improvement in lowest SpO_2_ and sleep efficiency, indicating enhanced airway patency and oxygenation during sleep. The increases in total sleep time and percentage of REM sleep, though the former was not statistically significant, suggest an overall subjective improvement in sleep quality.

The present study showed a 50.8% response rate. However, obese patients (BMI ≥ 25 kg/m²) were more likely to experience surgical failure (OR, 3.71). Severe OSA patients (AHI ≥ 30 events/hr) with TB narrowing also showed a trend toward poorer outcomes (OR, 2.23), although not statistically significant. These results suggest that, while endoscope‐guided coblation for TBR is generally effective, patients with higher BMI and more severe OSA may require additional or alternative therapeutic strategies—such as robot‐assisted TBR, HGNS, or PAP therapy—to achieve optimal outcomes, particularly when TB narrowing is present. Given the variability in outcomes based on patient characteristics such as BMI and severity of OSA, there is a need for continued innovation in surgical techniques and patient‐specific treatment plans.

We expected endoscopic coblation TBR to improve retroglossal narrowing in OSA patients and that it would be less invasive than robot‐assisted TBR or HGNS because coblation excises TB tissue with relatively lower temperature. The complication profile observed in this study aligns with previous reports on surgical interventions for OSA and the severity of complications was minimal. Postoperative pain was the most common but significantly diminished over time and no major complications such as massive bleeding or airway obstruction were observed. We suggest that the endoscope‐guided coblation might be a good surgical device to improve the TB narrowing in moderate to severe OSA subjects and for achieving favorable surgical outcomes with fewer complications even when performed in combination with nasal and palatal surgery. In addition, hyoid surgeries providing indirect tissue support have shown favorable outcomes in correcting hypopharyngeal collapse [30, 31], and can be incorporated into pattern‐based surgical protocols along with endoscopic coblation TBR. In patients with AHI > 15, protocols applying TBR for anteroposterior collapse, hyoidthyroidopexy for lateral collapse, and both procedures for concentric collapse have yielded favorable results [32].

The major limitation of this study is that the surgical outcomes of TBR were evaluated in combination with nasal and palatal surgeries, which may have confounded the specific effects of TBR alone. Additionally, the retrospective design and lack of a control group limit the ability to definitively establish causality, and the follow‐up period was relatively short, leaving longer‐term outcomes uncertain. Additionally, a direct comparison of the therapeutic outcomes of robot‐assisted TBR and HGNS was not included in our study. Since HGNS has not yet been approved by the Korea Food & Drug Administration, both endoscopic coblation techniques and robot‐assisted TBR are employed to correct TB narrowing in OSA patients in Korea [29]. We estimate that coblation techniques generally provide equivalent outcomes with fewer complications compared to robot‐assisted TBR [27, 28, 29, 33]. Nonetheless, there remains a demand for treatment not involving implantable devices and robot technologies. Future studies should include randomized controlled studies with extended follow‐up periods, and comparative studies using advanced techniques will be essential to overcome these limitations.

## 5 Conclusion

Endoscope‐guided coblation is an effective surgical option to improve airflow in the retroglossal area by resecting TB tissue, demonstrating significant improvements in key sleep parameters and a favorable safety profile as part of multilevel surgery in moderate to severe OSA patients.

## Author Contributions

Minju Kim and Hyun Jik Kim were involved in project conceptualization, data collection, formal analysis, writing and editing the manuscript. Siyeon Jin and Dongyoung Kim assisted with data collection, analysis and manuscript review. Doo Hee Han provided expertise and edits to final draft. Hyun Jik Kim contributed to the overall research supervision, as well as further editing and review. All authors contributed to manuscript revision, read and approved the submitted version.

## Conflicts of Interest

The authors declare no conflicts of interest.

## Data Availability

The data that support the findings of this study are available on request from the corresponding author.
